# Examining the roles of depression, pain catastrophizing, and self-efficacy in quality of life changes following chronic pain treatment

**DOI:** 10.1080/24740527.2022.2156330

**Published:** 2023-02-17

**Authors:** Landon T. Montag, Tim V. Salomons, Rosemary Wilson, Scott Duggan, Etienne J. Bisson

**Affiliations:** a Centre for Neuroscience Studies, Queen’s University, Kingston, Ontario, Canada; b Department of Psychology, Queen’s University, Kingston, Ontario, Canada; c School of Nursing, Queen’s University, Kingston, Ontario, Canada; d Chronic Pain Clinic, Kingston Health Sciences Centre, Kingston, Ontario, Canada; e Department of Anesthesiology and Perioperative Medicine, Queen’s University, Kingston, Ontario, Canada; f Department of Biomedical and Molecular Sciences, Queen’s University, Kingston, Ontario, Canada; g School of Rehabilitation Therapy, Queen’s University, Kingston, Ontario, Canada

**Keywords:** biopsychosocial, catastrophizing, chronic pain, depression, pain management, pain mechanisms, quality of life, coping

## Abstract

**Background:**

Adults with chronic pain have a lower quality of life (QOL) compared to the general population. Chronic pain requires specialized treatment to address the multitude of factors that contribute to an individual’s pain experience, and effectively managing pain requires a biopsychosocial approach to improve patients’ QOL.

**Aim:**

This study examined adults with chronic pain after a year of specialized treatment to determine the role of cognitive markers (i.e., pain catastrophizing, depression, pain self-efficacy) in predicting changes in QOL.

**Methods:**

Patients in an interdisciplinary chronic pain clinic (*N* = 197) completed measures of pain catastrophizing, depression, pain self-efficacy, and QOL at baseline and 1 year later. Correlations and a moderated mediation were completed to understand the relationships between the variables.

**Results:**

Higher baseline pain catastrophizing was significantly associated with increased mental QOL (*b* = 0.39, 95% confidence interval [CI] 0.141; 0.648) and decreased depression (*b* = −0.18, 95% CI −0.306; −0.052) over a year. Furthermore, the relationship between baseline pain catastrophizing and the change in depression was moderated by the change in pain self-efficacy (*b* = −0.10, 95% CI −0.145; −0.043) over a year. Patients with high baseline pain catastrophizing reported decreased depression after a year of treatment, which was associated with greater QOL improvements but only in patients with unchanged or improved pain self-efficacy.

**Conclusions:**

Our findings highlight the roles of cognitive and affective factors and their impact on QOL in adults with chronic pain. Understanding the psychological factors that predict increased mental QOL is clinically useful, because medical teams can optimize these positive changes in QOL through psychosocial interventions aimed at improving patients’ pain self-efficacy.

## RÉSUMÉ

**Contexte:** Les adultes souffrant de douleur chronique ont une qualité de vie inférieure à celle de la population en général. La douleur chronique nécessite un traitement spécialisé pour répondre à la multitude de facteurs qui contribuent à l’expérience de la douleur d’un individu. De plus, la prise en charge efficace de la douleur nécessite une approche biopsychosociale pour améliorer la qualité de vie des patients.

**Objectif:** Cette étude a examiné des adultes souffrant de douleur chronique après un an de traitement spécialisé pour déterminer le rôle des marqueurs cognitifs (c.-à-d. la catastrophisation de la douleur, la dépression, l’efficacité personnelle face à la douleur) dans la prévision des changements dans la qualité de vie.

**Méthodes:** Les patients d’une clinique interdisciplinaire de la douleur chronique (N = 197) ont effectué des mesures de la catastrophisation de la douleur, de la dépression, du sentiment d’efficacité personnelle face à la douleur, ainsi que de la qualité de vie au départ et un an plus tard. Des corrélations et une médiation modérée ont été effectuées pour comprendre les relations entre les variables.

**Résultats:** Une plus grande catastrophisation de la douleur au départ était significativement associée à une augmentation de la qualité de vie mentale (b = 0,39, intervalle de confiance à 95 % [IC] 0,141; 0,648) et à une diminution de la dépression (b = -0,18, IC à 95 % −0,306; −0,052) sur une année. En outre, la relation entre la catastrophisation de la douleur au départ et les changements en matière de dépression a été modérée par le changement dans le sentiment d’efficacité personnelle face à la douleur (b = − 0,10, IC à 95 % − 0,145; − 0,043) sur une année. Les patients démontrant une catastrophisation de la douleur élevée ont signalé une diminution de la dépression après un an de traitement, associée à de plus grandes améliorations dans la qualité de vie mais seulement chez les patients présentant un sentiment d’efficacité personnelle face à la douleur inchangée ou améliorée.

**Conclusions:** Nos résultats mettent en évidence le rôle des facteurs cognitifs et affectifs et leur effet sur la qualité de vie chez les adultes souffrant de douleur chronique. Il est cliniquement utile de comprendre les facteurs psychologiques qui prédisent une augmentation la qualité de vie mentale, car les équipes médicales peuvent optimiser ces changements positifs dans la qualité de vie par des interventions psychosociales visant à améliorer le sentiment d’efficacité personnelle des patients face à la douleur.

## Introduction

One in five adult Canadians are afflicted with chronic pain, a widespread and debilitating condition.^1^ The chronic pain experience often includes complex interactions between physiological, emotional, cognitive, social, and environmental factors,^2^ and psychological factors can heavily influence symptom presentation and prognosis.^2,3^ Therefore, specialized pain treatment, as offered in interdisciplinary chronic pain clinics and programs, addresses biological, psychological, and social factors that contribute to an individual’s pain experience to optimize pain relief and coping. Specialized pain treatment often focuses on improving patients’ function and quality of life (QOL), rather than pain elimination. Patients treated in an interdisciplinary pain clinic have demonstrated significant improvements in QOL after 6 months and a significant decrease in pain severity after 1 year.^4^

Broadly defined as one’s perceived overall well-being, QOL is an important target in pain management. When pain is not effectively treated and/or managed, it has a significant negative impact on all dimensions of QOL. In fact, adults with chronic pain have significantly lower QOL compared to the general population^5,6^ but also lower than adults with cancer, heart disease, or diabetes.^7^ As such, we wanted to better understand the factors that drive improvements in QOL in adults with chronic pain. It is often thought that patients’ QOL would simply improve by providing pain relief; however, a study examining patients with low back pain failed to find a significant relationship between pain relief and improvements in QOL that exceeded 2 months.^8^ Additionally, Kovacs et al.^9^ found that QOL was predicted by disability, not pain severity. Psychological factors such as pain catastrophizing,^6,10^ depression,^11^ and self-efficacy^12,13^ were also found to be better predictors of QOL than pain intensity. Pain catastrophizing (a maladaptive cognitive process of exaggerated, negative reactions to expected or actual painful experiences^14,15^) was found to be independently associated with QOL^10,13,16^ and the strongest predictor of QOL compared to pain intensity and demographic variables^6^ in adults with chronic pain. High pain catastrophizing has been associated with poor treatment outcomes,^16^ and thus we were interested in examining the negative relationship between pain catastrophizing and QOL to determine a mechanism by which it can be reversed. In clinical practice, it is vital to determine how to improve QOL outcomes for patients with high pain catastrophizing at the point of intake for specialized pain care.

Because pain catastrophizing is a strong predictor of reduced QOL, it is important for clinicians to better understand what drives this relationship. Depression may mediate because pain catastrophizing is a strong predictor of depression^17,18^ and depression is associated with low perceived QOL.^11,19,20^ However, to our knowledge, no studies have examined this mediating effect. It is also important to investigate what might drive the relationship between pain catastrophizing and depression. One domain of pain catastrophizing is helplessness, and the degree to which a person catastrophizes is a function of their feelings of control (or lack thereof) over their pain. Patients with higher levels of pain self-efficacy (an individual’s beliefs about their ability to successfully cope with pain and/or related negative emotions, to complete daily tasks, and to implement pain management strategies^21^) are less likely to engage in pain catastrophizing.^22–24^ Adaptive cognitive processes (e.g., pain self-efficacy) contribute to resilient pain coping,^25^ and patients with higher self-efficacy report less pain,^26–30^ depression,^26,29,31–33^ disability,^26–28,34,35^ affective distress,^28,36^ and catastrophizing.^12,29,37^ Because an individual’s sense of control over their pain is a key part of pain catastrophizing, self-efficacy may be a potential moderator by which patients improve their pain catastrophizing and depressive symptoms. In fact, pain self-efficacy has been found to serve as a moderating variable between pain intensity and depression and between pain intensity and pain catastrophizing in adults with chronic pain.^37^ Further research is required to determine pain self-efficacy’s role in moderating the relationship between pain catastrophizing and depression directly. Clinically, it is also important to assess the role of pain self-efficacy, because it is often a targetable outcome in self-management programs for chronic pain.

Few studies have examined the psychological factors that predict changes in adult patients’ QOL over a year of specialized chronic pain treatment.^38,39^ Examining these cognitive markers and developing a better understanding of the mechanism by which QOL can be improved could lead to more targeted treatments for chronic pain. Therefore, the goal of this study was to determine the role of pain catastrophizing, depression, and pain self-efficacy in predicting changes in QOL in adults with chronic pain after a year of specialized treatment. First, we investigated how patients’ pain catastrophizing at intake into an interdisciplinary chronic pain clinic related to their change in QOL over a year and determined whether their change in depression was a mediator of this relationship. Second, we examined whether the mediating role of their change in depression varied as a function of their change in pain self-efficacy. We predicted that lower baseline pain catastrophizing would be related to improved QOL over a year and that this relationship was explained by the mediating effect of decreased depression over the year (Hypothesis 1). However, we predicted that this relationship would only occur for patients with pain self-efficacy that increased over the year (Hypothesis 2).

## Methods

### Study Design and Data Source

This observational study used patient-reported data from the Kingston Health Sciences Centre (KHSC) Chronic Pain Registry collected from November 2017 to May 2021. This chronic pain database included sociodemographic information and biopsychosocial outcomes from patients attending the KHSC Chronic Pain Clinic (KHSC-CPC) in Kingston, Ontario, Canada. The KHSC-CPC is a publicly funded outpatient service providing comprehensive pain management to adults living with chronic pain throughout Southeastern Ontario. Using an interdisciplinary approach, a variety of treatments are offered in form of group-based interventions, one-on-one consultation with specific disciplines, medication management and optimization, and interventional pain management procedures. The clinical team includes four anesthesiologists, one neurosurgeon, four nurse practitioners, three registered nurses, three registered practical nurses, two physiotherapists, two occupational therapists, one psychologist, and two social workers, supported by administrative staff and a clinical research coordinator.

Patients seeking care at the KHSC-CPC completed an initial self-report questionnaire package before their first assessment. Patients were informed that their data would be used for clinical services and for research purposes, should they choose to provide written consent for research involvement. It was clarified to patients that involvement in research was voluntary and would not affect the health care they received at the clinic. Patients who did not provide consent for their information to be used for research were excluded from this study. Patients attending the KHSC-CPC were also asked to complete a self-report questionnaire at every subsequent visit with a physician or nurse practitioner. This study was approved by the Queen’s University Health Sciences and Affiliated Teaching Hospital’s Research Ethics Board (ANA-336-18-6023876).

### Measures

**Figure 1.**
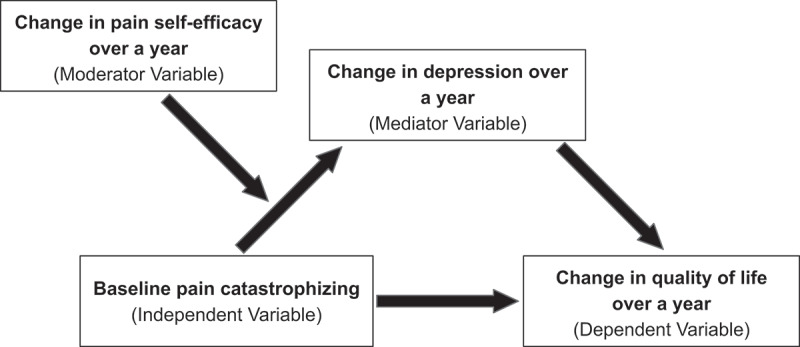
The proposed moderated mediation model.

Baseline questionnaires (at intake into the clinic) and those completed 1 year later at a follow-up appointment are a component of usual data collection and were used in all analyses for this study. The proposed moderated mediation model is presented in Figure 1.

Pain catastrophizing was measured using the Pain Catastrophizing Scale Short Form (PCS-6).^40^ PCS-6 is a six-item measure where participants indicate the frequency with which they experienced pain-related thoughts and feelings on a 5-point scale (0 = *not at all*, 4 = *all the time*). PCS-6 has good reliability.^41,42^ For the analyses, baseline total PCS-6 scores were obtained by summing the items. Higher scores indicated increased (worse) pain catastrophizing.

Quality of life was measured using the Short Form-12 Health Survey (SF-12v2).^43^ SF-12v2 is a 12-item measure examining eight health domains. The SF-12v2 is scored by summing the appropriate items into two separate summary scores, the physical component summary and the mental component summary, indicating physical QOL and mental QOL, respectively. SF-12v2 has good reliability.^44^ To obtain the change in physical and mental QOL, we subtracted each participant’s component score at baseline from their component score at follow-up. Higher scores indicated improved physical or mental QOL.

Depression was measured using the Patient Health Questionnaire (PHQ-9).^45^ PHQ-9 is a 9-item measure assessing the degree of depression severity over the past 2 weeks on a 4-point scale (0 = *not at all*, 3 = *nearly every day*). PHQ-9 has good reliability.^45^ Total PHQ-9 scores were obtained by summing the items. To obtain the change in depression, we subtracted each participant’s total score at baseline from their total score at follow-up. Higher scores indicated increased (more severe) depression.

Pain self-efficacy was measured using the Short Form Pain Self-Efficacy Scale (PSEQ-2).^46^ PSEQ-2 is a two-item measure where participants indicate the degree to which they believe they can complete daily tasks on a 7-point scale (0 = *not at all confident*, 6 = *completely confident*). PSEQ-2 has good reliability.^46^ Total PSEQ-2 scores were obtained by summing the items. To obtain the change in pain self-efficacy, we subtracted each participant’s score at baseline from their score at follow-up. Higher scores indicated improved pain self-efficacy.

### Statistical Analyses

Pearson correlations assessed the relationships between the variables in the study. Correlations were considered weak if the correlation coefficient was <0.4, moderate if between 0.4 and 0.7, and strong if ≥0.7.^47^ To test our hypotheses, we performed a statistical mediation and moderated mediation analyses using the lavaan package^48^ in R.^49^ We used a statistical mediation model because we wanted to understand the factors that relate to the key outcome of patients’ QOL. Acknowledging the inferential limitations of mediation models^50,51^ (specifically that they do not determine causality), we will for simplicity herein refer to statistical mediation as mediation, without implying causality. In both the mediation and moderated mediation analyses, 10,000 bootstrapped estimates were used for the construction of 95th percentile corrected confidence intervals (CIs) for the conditional indirect effects.^52,53^ In the moderated mediation, these CIs were used to assess the significance of the indirect effects at specific values of the moderator (the change in pain self-efficacy) using the “pick-a-point” approach.^54,55^ The centered mean and a standard deviation above and below the centered mean of pain self-efficacy were used to represent unchanged, increased, and decreased pain self-efficacy, respectively. In both the mediation and moderated mediation analyses, the reported *b*s are standardized regression coefficients. The criterion for statistical significance was the absence of zero within the confidence intervals. Although mediation and moderated mediation analyses were performed with either physical or mental QOL change score as the outcome, no significant mediation effects were found with patients’ change in physical QOL scores, and thus only the findings using patients’ change in mental QOL are reported. To ensure that the model was not driven by patients’ change in pain severity, we also ran the model controlling for this. Because similar results were found when running both models, we will hereafter only report the results of the simpler and more naturalistic model, which did not control for the change in pain severity.

## Results

### Participants

The study sample consisted of 251 eligible participants who had chronic pain, were ≥18 years old, and had an initial and 1-year appointment completed at the KHSC-CPC. Because 54 participants did not fully complete the questionnaires, they were excluded from the analyses. The final sample included in the analyses consisted of 197 participants with an average age of 51.53 years (SD 15.07, range = 18–91). Most participants were women (62.9%, *n* = 124), nonsmokers (60.9%, *n* = 92), living in a household with other adult(s) (56.9%, *n* = 107), and completed college or a high level of education (56.0%, *n* = 103). The study sample included adults with neuropathic or nonneuropathic pain (69.0%, *n* = 136) who reported on average 3.61 pain sites (SD 2.15, range = 0–9). The three most reported pain sites were chronic low back pain (74.6%, *n* = 147), lower limb pain (60.9%, *n* = 120), and upper limb pain (53.8%, *n* = 106). Participants had on average 4.11 (SD 0.88, range = 2–5) visits in their first year as a patient at KHSC-CPC. On average, participants had significantly improved physical QOL, decreased pain catastrophizing, and decreased depressive symptoms after 1 year of treatment (Table 1).

**Table 1.** Mean and SD for the study variables (*N* = 195) at baseline and 1-year follow-up.

	Baseline		Follow-up		Change scores	
Variable	Mean	SD	Mean	SD	Mean	SD
Pain catastrophizing	15.30	5.13	12.15	6.31	−3.15***	5.64
Mental QOL	39.77	12.37	41.21	11.82	1.44	10.48
Physical QOL	30.91	9.63	33.19	9.82	2.28*	7.90
Depression	12.30	6.11	10.29	6.38	−2.01**	5.38
Pain self-efficacy	7.01	3.27	7.09	2.95	0.09	2.75

Note: *P < 0.05, **P < 0.01, ***P < 0.001 and indicates a significant difference between baseline and follow-up mean scores.

### Correlation Analyses

The correlation coefficients between the study variables are presented in Table 2. We found a significant, albeit weak, correlation between baseline pain catastrophizing and the change in mental QOL (*r* = 0.20) as well as the change in depression (*r* = −0.21). The change in depression and change in mental QOL had a significant, moderate correlation (*r* = −0.60). There was no significant correlation between baseline pain catastrophizing and the change in pain self-efficacy (*r* = 0.10). The change in pain catastrophizing and change in mental and physical QOL was significant and negatively correlated (*r* = −0.28, *P* < 0.001, *n* = 194 and *r* = −0.33, *P* < 0.001, *n* = 194, respectively). Patients’ age and sex were not correlated with any of the study variables.

**Table 2.** Pearson correlation coefficients for the study variables (*N* = 195).

Variable	1	2	3	4	5	6	7
1. Baseline pain catastrophizing							
2. Baseline physical QOL	−0.19**						
3. Baseline mental QOL	−0.52***						
4. Baseline depression	0.61***	−0.29***	−0.75***				
5. Change in physical QOL	0.03	−0.39***	0.25***	−0.06			
6. Change in mental QOL	0.21**	−0.01	−0.48***	0.32***			
7. Change in depression	−0.20**	0.13	0.18**	−0.39***	−0.15*	−0.62***	
8. Change in pain self-efficacy	0.10	−0.11	−0.18*	0.18*	0.18*	0.34***	−0.30***

Note: *P < 0.05, **P < 0.01, ***P < 0.001.

### Mediation Analyses

First, we tested whether baseline pain catastrophizing was related to patients’ change in QOL after 1 year. This relationship was only significant for mental QOL, such that higher (or more severe) pain catastrophizing was associated with greater improvements in mental QOL (*b* = 0.39; *z* = 3.00; 95% CI 0.141, 0.648). Next, we examined the indirect effect by testing whether the patients’ change in depression mediated the effect of baseline pain catastrophizing on the change in QOL. Results from a joint significance test^56^ indicated that the indirect effect was only significant for mental QOL, such that more severe baseline pain catastrophizing was associated with decreased depression over the year (*b* = −0.18; *z* = −2.75; 95% CI −0.306, −0.052) and decreased depression was associated with increased mental QOL while controlling for pain catastrophizing (*b* = −1.16; *z* = −10.61; 95% CI −1.365, −0.931). The effect of pain catastrophizing on the change in mental QOL after controlling for change in depression was not significant (*b* = 0.19; *z* = 1.61; 95% CI −0.039, 0.418).

### Moderated Mediation Analyses

**Figure 2.**
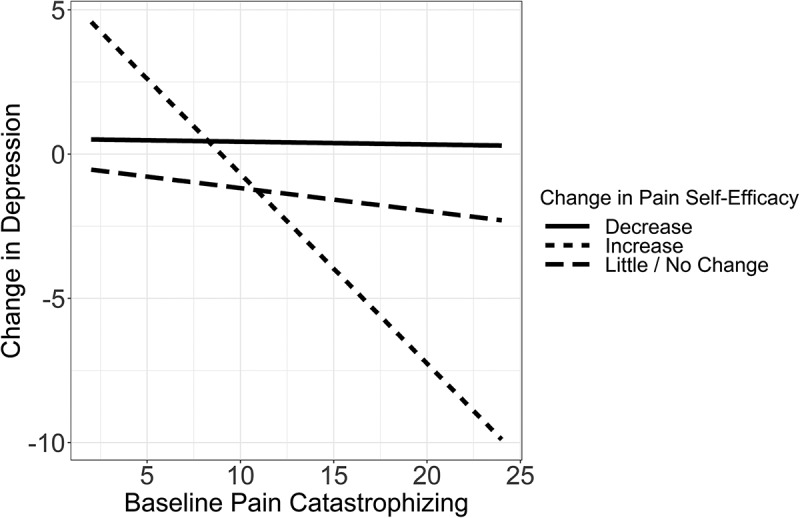
The moderation effect of the change in pain self-efficacy between baseline pain catastrophizing and change in depression. When pain self-efficacy decreased or was unchanged, there was no relationship between baseline pain catastrophizing and change in depression. However, there was a significant relationship between pain catastrophizing and the change in depression when pain self-efficacy increased. Patients with lower baseline pain catastrophizing had more severe (or worsening) depression after a year. Conversely, those with high baseline pain catastrophizing had decreased (or improved) depression after a year.

The moderated mediation model (Figure 1) was assessed using Hayes’ model 7.^57^ We tested the conditional indirect effect of the change in pain self-efficacy on the relationship between baseline pain catastrophizing and the change in mental QOL that occurs through the change in depression. Specifically, we assessed whether the change in pain self-efficacy moderated the relationship between pain catastrophizing and the change in depression. Each variable was continuous, and centered values were used for pain catastrophizing, change in depression, and change in pain self-efficacy. The moderation effect is presented in Figure 2.

We found that the relationship between baseline pain catastrophizing and the change in depression was moderated by patients’ change in pain self-efficacy (*b* = −0.10; *z* = −3.59; 95% CI −0.145, −0.043). Altogether, the mediation was moderated, such that the indirect effect of pain catastrophizing on the change in mental QOL through the change in depression depended on the change in pain self-efficacy (*b* = 0.11; *z* = 3.25; 95% CI 0.046, 0.177). Overall, the model accounted for 15% of the variance in the change in depression (*R*^2^ = 0.146) and 38% in the change in mental QOL (*R*^2^ = 0.378).

The pick-a-point approach was used to examine the significant relationship between pain catastrophizing and change in depression at one standard deviation below and above as well as at mean levels of pain self-efficacy. One hundred thirty-seven (69.5%) participants had unchanged or little change in their pain self-efficacy, 25 (12.7%) participants had decreased, and 35 (17.8%) participants had increased pain self-efficacy after a year. The indirect effect of pain catastrophizing on the change in mental QOL through change in depression was significant among patients with unchanged (*b* = 0.22; *z* = 2.56; 95% CI 0.060, 0.389) and increased (*b* = 0.51; *z* = 3.47; 95% CI 0.230, 0.799) pain self-efficacy but was not significant among patients with decreased (*b* = −0.09; *z* = −0.96; 95% CI −0.286, 0.089) pain self-efficacy. Altogether, a significant relationship between pain catastrophizing and change in depression was found across multiple levels of pain self-efficacy. As pain self-efficacy improved, the beta values of the indirect conditional effects increased. These results suggest that the relationship between pain catastrophizing and change in depression becomes stronger as pain self-efficacy improves.

## Discussion

This study demonstrates that the relationship between baseline pain catastrophizing and the change in mental QOL after a year is mediated by the patients’ change in depression. Furthermore, the relationship between baseline pain catastrophizing and the change in depression (the mediation effect) depends on the patients’ change in pain self-efficacy. Specifically, patients with high baseline pain catastrophizing reported decreased depression, and this association was greater as pain self-efficacy increased. The results partly support Hypothesis 1 because higher pain catastrophizing scores at baseline were associated with improved mental QOL scores over a year, and this relationship was mediated by decreased depression over the year. The results also partly support Hypothesis 2 because this mediated relationship was moderated by increased and unchanged pain self-efficacy. Note that because information about specific treatment(s) and intervention(s) received by participants in our sample was not available, clinical implications of our findings are further discussed based on previous literature.

It was surprising that the mediation analyses were only significant for mental QOL rather than both physical and mental QOL, because higher pain catastrophizing has been found to have a direct negative association on depression, which negatively impacts physical and mental QOL.^58^ In our study, the mediation using mental QOL was found to be significant because pain catastrophizing, depression, and self-efficacy are variables representative of psychological functioning, and significant correlations were found in our sample between these variables and baseline mental QOL (as seen in Table 2). On the other hand, in our sample, physical QOL may have correlated more highly with physical functioning, such as pain severity and pain interference with daily activities, and thus changes in physical QOL may have depended more on changes in the manifestation of pain symptoms.

In this study, we have determined a mechanism by which mental QOL can be improved over a year of specialized chronic pain treatment. Firstly, we found that patients with the greatest improvement in mental QOL over a year were those with high baseline pain catastrophizing. This is supported by previous literature because individuals not receiving specialized pain treatment have high pain catastrophizing, and higher pain catastrophizing is related to strong fear avoidance beliefs, thus negatively impacting physical and mental QOL over time.^58,59^ Our findings suggest that patients with high pain catastrophizing can improve their QOL by receiving specialized pain treatment. In fact, Gilliam et al.^60^ examined the effects of a 3-week cognitive behavioral therapy program for adults with chronic pain and found that patients who had improved pain catastrophizing also had significant improvements in mental QOL compared to those with unchanged pain catastrophizing.^60^

To explore a mechanism by which changes in mental QOL can be related to baseline pain catastrophizing, we examined patients’ change in depression over the year. We found that the change in depression was a significant mediator of this relationship, such that higher baseline pain catastrophizing was significantly associated with decreased depression over the year, which in turn was related to improved mental QOL. It is likely that people with high baseline pain catastrophizing had the most room to improve on their depression and mental QOL scores, as supported with the high correlations of these measures at baseline. This is also supported by the literature, because improvements in pain catastrophizing precede changes in pain-related outcomes in patients receiving multidisciplinary chronic pain treatment.^61,62^ Additionally, we found that the change in depression had a significant negative association with mental QOL while controlling for pain catastrophizing. This finding aligns with existing evidence that improvements in depression predict better pain outcomes and increased QOL.^63,64^ This can be applied clinically, because patients with high pain catastrophizing can be streamlined into interventions to improve their depressive symptoms, which will indirectly improve their QOL. For instance, Craner et al.^65^ found that after undergoing a 3-week multidisciplinary pain rehabilitation program, adults with chronic pain had significantly decreased pain catastrophizing, which significantly mediated the improvement in depressive symptoms. Psychological interventions (e.g., acceptance and mindfulness-based treatments) for chronic pain have also been found to significantly reduce catastrophizing beliefs^66^ and depression,^67^ as well as improve pain self-efficacy^66^ and health-related QOL.^67^ A meta-analysis of acceptance- and mindfulness-based treatments for chronic pain found moderate effect sizes for depression and QOL 2 to 6 months posttreatment.^68^

To further elucidate how a greater reduction in depression over the year was related to high baseline pain catastrophizing, we assessed whether the mediation effect was moderated by another variable. We examined self-efficacy because it has been found to serve as a protective factor against depression in those with chronic pain.^32,33^ We found that pain self-efficacy moderated the association between pain catastrophizing and change in depression in patients with unchanged or increased pain self-efficacy, and the relationship became more positive as pain self-efficacy increased. However, this relationship was not significant for patients with decreased pain self-efficacy. This finding is partially supported by the literature, because Cheng et al.^37^ found that pain self-efficacy moderated the relationship between pain intensity and depressive symptoms, as well as the relationship between pain intensity and pain catastrophizing. These findings suggest that increasing levels of pain self-efficacy may reduce depressive symptoms by reducing pain catastrophizing.^37^ Similarly, the findings of the current study support that unchanged or improved pain self-efficacy may be an important mechanism in improving the mental QOL of patients with chronic pain. However, the cross-sectional nature of this study prevents strong conclusions.

This study highlights the importance of identifying baseline psychosocial characteristics in patients that can predict changes in their QOL long term. By evaluating the mechanism by which adult patients’ mental QOL increases over a year of specialized treatment, we better understand the importance of improving pain self-efficacy in those with high pain catastrophizing at intake into an interdisciplinary pain clinic. In fact, improving pain self-efficacy and decreasing pain catastrophizing have been found to prolong the positive effects of specialized pain treatment.^29^ By identifying these key characteristics at baseline, patients can be streamlined into psychological interventions that target these psychosocial variables, thus resulting in improved QOL in a shorter period of time. These psychosocial variables may also be baseline indicators that can be used to predict the likelihood of patients’ future successes in certain psychological interventions (e.g., targeting pain catastrophizing, improving pain self-efficacy).

Some study limitations should be noted. First, our findings cannot be linked to a specific treatment or type of interventions that patients received over the year, because these data were not available in the KHSC Chronic Pain Registry. Thus, it is unclear which treatments/interventions led to improvements in depressive symptoms, pain self-efficacy, and mental QOL. Note that for this reason, our earlier discussion on clinical implications and which interventions led to changes in pain catastrophizing, depressive symptoms, self-efficacy, and mental QOL was based on previous literature, rather than recommendations based directly from our findings. Our findings suggest that pain treatment focusing on pain self-efficacy would be best for patients with high pain catastrophizing to improve QOL, but this needs to be confirmed with further investigation. Second, this study used a correlational design; thus, we cannot make conclusions about the causal relationships between the study variables. Further, because there are only two time points in this study, reverse causality cannot be ruled out.

## Conclusion

The results of this study highlight the roles of cognitive and affective factors and their impact on mental QOL in adults with chronic pain. It was found that patients’ change in depression mediated the relationship between baseline pain catastrophizing and the change in mental QOL after a year. It was further determined that this mediation effect was moderated by patients’ change in pain self-efficacy, as long as their self-efficacy remained unchanged or improved. Understanding the psychological factors that predict increased mental QOL is clinically useful, because medical teams may be able to optimize these positive changes in QOL through psychosocial interventions aimed at improving patients’ pain self-efficacy. Our findings suggest that individuals with high pain catastrophizing at intake into a specialized pain clinic should be targeted for self-management programs/interventions that target improvement in self-efficacy.
